# Supplementary material for: All trans-retinoic acid modulates hyperoxia-induced suppression of NF-kB-dependent Wnt signaling in alveolar A549 epithelial cells
Source: PLoS One. 2022 Aug 10;17(8):e0272769. doi: 10.1371/journal.pone.0272769 (PMC9365139; doi:10.1371/journal.pone.0272769)
Supplement: S1 Table — (DOCX) [file pone.0272769.s001.docx]

S1 Table

*ANOVA Summary Table for cell numbers in the 24-hour time point*

|  | SS | DF | MS | F (DFn, DFd) | P value |
| --- | --- | --- | --- | --- | --- |
| Interaction | 849.7 | 3 | 283.2 | F (3, 12) = 3.650 | P=0.0445 |
| ATRA concentration | 6443 | 3 | 2148 | F (1.597, 6.388) = 27.67 | P=0.0009 |
| Oxygen exposure | 533.0 | 1 | 533.0 | F (1, 4) = 3.253 | P=0.1456 |
| Replicate | 655.4 | 4 | 163.9 | F (4, 12) = 2.111 | P=0.1422 |
| Residual | 931.3 | 12 | 77.61 |  |  |

_____________________________________________________________________________________

SS = Sum-of-squares, DF = Degrees of freedom, MS = Mean squares, F = F-statistic, DFn = Degrees of freedom in the numerator, DFd = Degrees of freedom in the denominator.
