# Supplementary material for: All trans-retinoic acid modulates hyperoxia-induced suppression of NF-kB-dependent Wnt signaling in alveolar A549 epithelial cells
Source: PLoS One. 2022 Aug 10;17(8):e0272769. doi: 10.1371/journal.pone.0272769 (PMC9365139; doi:10.1371/journal.pone.0272769)
Supplement: S2 Table — (DOCX) [file pone.0272769.s002.docx]

S2 Table

*ANOVA Summary Table for cell numbers in the 48-hour time point*

|  | SS | DF | MS | F (DFn, DFd) | P value |
| --- | --- | --- | --- | --- | --- |
| Interaction | 29446 | 3 | 9815 | F (3, 12) = 60.68 | P<0.0001 |
| ATRA concentration | 48368 | 3 | 16123 | F (1.566, 6.264) = 99.67 | P<0.0001 |
| Oxygen exposure | 254719 | 1 | 254719 | F (1, 4) = 1035 | P<0.0001 |
| Replicate | 984.3 | 4 | 246.1 | F (4, 12) = 1.521 | P=0.2576 |
| Residual | 1941 | 12 | 161.8 |  |  |

_____________________________________________________________________________________

SS = Sum-of-squares, DF = Degrees of freedom, MS = Mean squares, F = F-statistic, DFn = Degrees of freedom in the numerator, DFd = Degrees of freedom in the denominator.
