# Supplementary material for: All trans-retinoic acid modulates hyperoxia-induced suppression of NF-kB-dependent Wnt signaling in alveolar A549 epithelial cells
Source: PLoS One. 2022 Aug 10;17(8):e0272769. doi: 10.1371/journal.pone.0272769 (PMC9365139; doi:10.1371/journal.pone.0272769)
Supplement: S3 Table — (DOCX) [file pone.0272769.s003.docx]

S3 Table

*ANOVA Summary Table for cell numbers in the 72-hour time point*

|  | SS | DF | MS | F (DFn, DFd) | P value |
| --- | --- | --- | --- | --- | --- |
| Interaction | 98077 | 3 | 32692 | F (3, 12) = 15.14 | P=0.0002 |
| ATRA concentration | 173523 | 3 | 57841 | F (1.323, 5.293) = 26.79 | P=0.0024 |
| Oxygen exposure | 856170 | 1 | 856170 | F (1, 4) = 5480 | P<0.0001 |
| Replicate | 625.0 | 4 | 156.2 | F (4, 12) = 0.07237 | P=0.9892 |
| Residual | 25908 | 12 | 2159 |  |  |

_____________________________________________________________________________________

SS = Sum-of-squares, DF = Degrees of freedom, MS = Mean squares, F = F-statistic, DFn = Degrees of freedom in the numerator, DFd = Degrees of freedom in the denominator.
