# Supplementary material for: All trans-retinoic acid modulates hyperoxia-induced suppression of NF-kB-dependent Wnt signaling in alveolar A549 epithelial cells
Source: PLoS One. 2022 Aug 10;17(8):e0272769. doi: 10.1371/journal.pone.0272769 (PMC9365139; doi:10.1371/journal.pone.0272769)
Supplement: S4 Table — Following RNA-seq analysis of A549 cells treated with hyperoxia, enrichment analysis was performed with the topGO package. The top 20 terms associated with biological processes are listed. (DOCX) [file pone.0272769.s004.docx]

| GO.ID | Term | elim Fisher pValue |
| --- | --- | --- |
| GO:0051301 | cell division | 9.10E-26 |
| GO:0000184 | nuclear-transcribed mRNA catabolic proce... | 1.30E-25 |
| GO:0006614 | SRP-dependent cotranslational protein ta... | 3.20E-24 |
| GO:0019083 | viral transcription | 1.30E-22 |
| GO:0006413 | translational initiation | 1.80E-20 |
| GO:0016032 | viral process | 6.10E-15 |
| GO:0050821 | protein stabilization | 2.00E-13 |
| GO:0043488 | regulation of mRNA stability | 3.80E-13 |
| GO:1900034 | regulation of cellular response to heat | 2.70E-12 |
| GO:0032436 | positive regulation of proteasomal ubiqu... | 1.40E-11 |
| GO:0071712 | ER-associated misfolded protein cataboli... | 5.60E-11 |
| GO:0043687 | post-translational protein modification | 9.50E-10 |
| GO:0010389 | regulation of G2/M transition of mitotic... | 1.10E-09 |
| GO:0000122 | negative regulation of transcription by ... | 1.60E-09 |
| GO:0006298 | mismatch repair | 2.10E-09 |
| GO:0007052 | mitotic spindle organization | 2.30E-09 |
| GO:1901796 | regulation of signal transduction by p53... | 3.80E-09 |
| GO:0006260 | DNA replication | 5.40E-09 |
| GO:0000082 | G1/S transition of mitotic cell cycle | 2.30E-08 |
| GO:0006406 | mRNA export from nucleus | 4.00E-08 |
