# Supplementary material for: All trans-retinoic acid modulates hyperoxia-induced suppression of NF-kB-dependent Wnt signaling in alveolar A549 epithelial cells
Source: PLoS One. 2022 Aug 10;17(8):e0272769. doi: 10.1371/journal.pone.0272769 (PMC9365139; doi:10.1371/journal.pone.0272769)
Supplement: S7 Table — Following RNA-seq analysis of A549 cells treated with hyperoxia, enrichment analysis was performed with the topGO package. The top 20 terms associated with biological processes are listed. (DOCX) [file pone.0272769.s007.docx]

| **GO.ID** | **Term** | **elim Fisher p-value** |
| --- | --- | --- |
| GO:0043312 | neutrophil degranulation | 7.00E-11 |
| GO:1904290 | negative regulation of mitotic DNA damag... | 8.20E-11 |
| GO:0036149 | phosphatidylinositol acyl-chain remodeli... | 8.80E-11 |
| GO:0021819 | layer formation in cerebral cortex | 1.20E-10 |
| GO:0038063 | collagen-activated tyrosine kinase recep... | 1.30E-10 |
| GO:0061302 | smooth muscle cell-matrix adhesion | 2.80E-09 |
| GO:0036151 | phosphatidylcholine acyl-chain remodelin... | 1.10E-08 |
| GO:0090207 | regulation of triglyceride metabolic pro... | 4.90E-08 |
| GO:0071364 | cellular response to epidermal growth fa... | 6.10E-08 |
| GO:0043066 | negative regulation of apoptotic process | 5.60E-07 |
| GO:0006606 | protein import into nucleus | 1.30E-06 |
| GO:0031638 | zymogen activation | 1.40E-06 |
| GO:0043065 | positive regulation of apoptotic process | 2.60E-06 |
| GO:0055114 | oxidation-reduction process | 3.10E-06 |
| GO:0010715 | regulation of extracellular matrix disas... | 4.20E-06 |
| GO:0010667 | negative regulation of cardiac muscle ce... | 5.80E-06 |
| GO:0002064 | epithelial cell development | 6.00E-06 |
| GO:0072659 | protein localization to plasma membrane | 6.10E-06 |
| GO:0001649 | osteoblast differentiation | 6.80E-06 |
| GO:0034775 | glutathione transmembrane transport | 8.40E-06 |
