# Supplementary figures and images for: All trans-retinoic acid modulates hyperoxia-induced suppression of NF-kB-dependent Wnt signaling in alveolar A549 epithelial cells
Source: PLoS One. 2022 Aug 10;17(8):e0272769. doi: 10.1371/journal.pone.0272769 (PMC9365139; doi:10.1371/journal.pone.0272769)

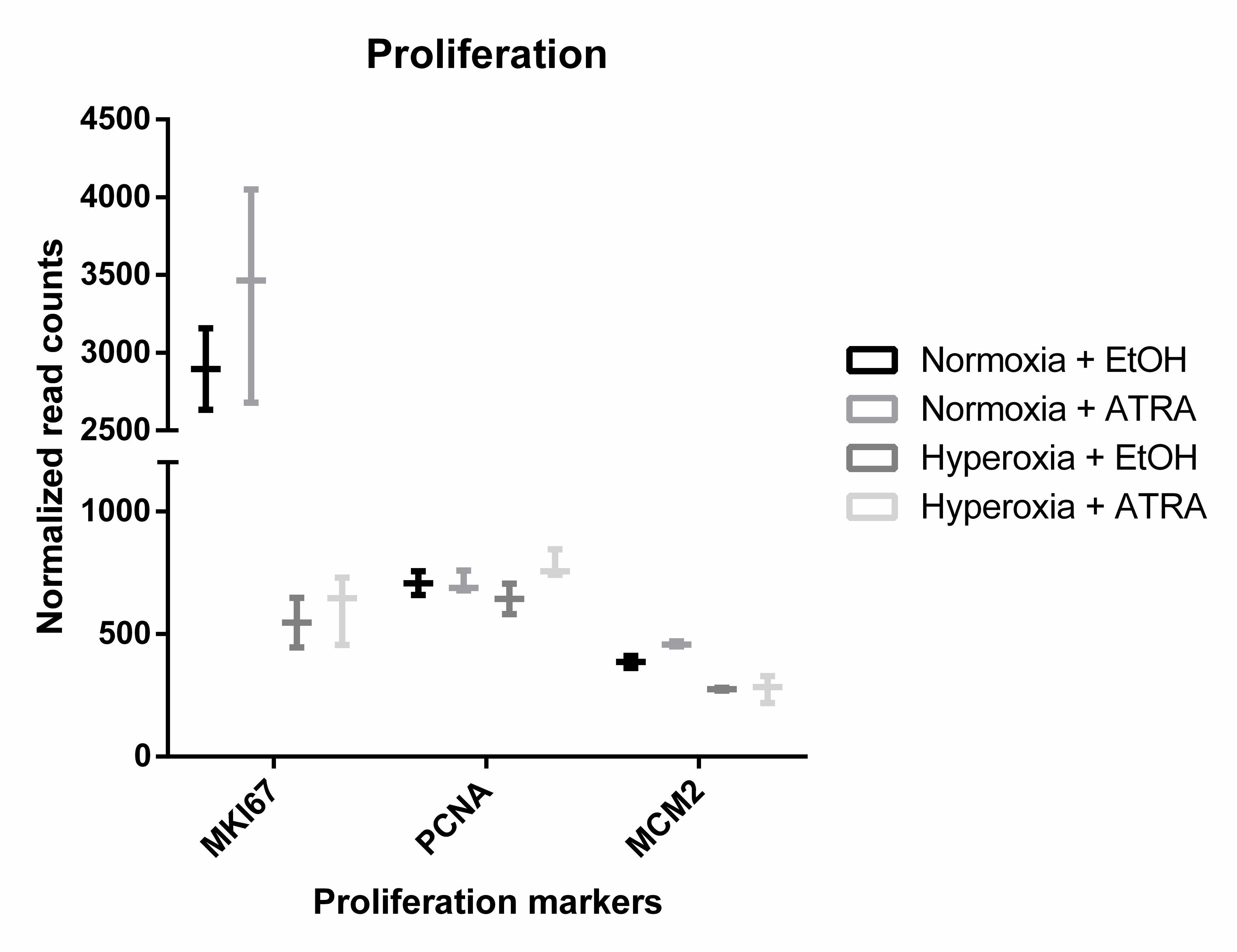

Supplement: S1 Fig — Following DESeq analysis of the RNA-seq results, normalized counts for transcripts of proliferation markers Ki67, PCNA, and MCM-2 were mined. (TIF) [file pone.0272769.s008.tif]

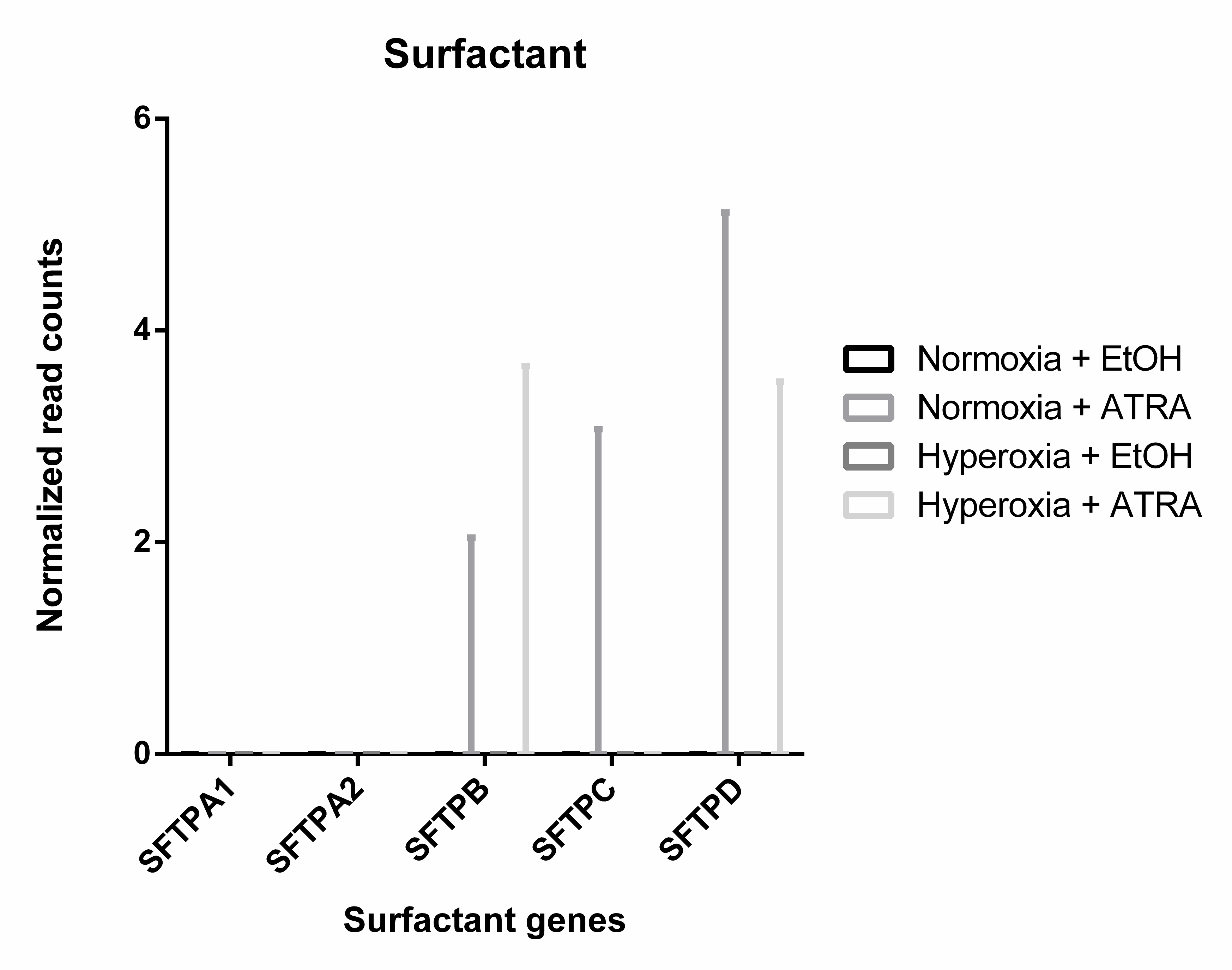

Supplement: S2 Fig — Following DESeq analysis of the RNA-seq results, normalized counts for transcripts of surfactant genes SFTPA1, SFTPA2, SFTPB, SFTPC, and SFTPD were mined. At least two samples per condition had zero counts for each gene. (TIF) [file pone.0272769.s009.tif]
